# 

**Published:** 1889-05

**Authors:** 


### CONTENTS.

### COMMUNICATIONS.

Name and Describe the Causes of Dental Caries and the Vital, Structural, and Chemical Changes Occuring During this Process...... 213
What Variations in the manner of Using the Teeth Are Exhibited by the Various Animals Including Man...... 221
The Peridental Membrane...... 228
A Criticism...... 230
Pulp Stones...... 232
Scurvy...... 233
Monthly Digest...... 236
Dental College Commencements...... 244

### SELECTIONS.

What Kills the Children...... 253
Effects of Overwork...... 256
States May Regulate Medical Practice...... 257
Brain Workers...... 258
Transactions of the Ninth International Medical Congress...... 259

### EDITORIAL.

Dental Society Meetings...... 260
Prehistoric Dentistry—Bridge Work...... 261
Special Notice...... 261
Alumni Notice...... 263
Post-Graduate School...... 264

